# Autophagy maintains the stemness of ovarian cancer stem cells by FOXA2.

**DOI:** 10.1186/s13046-017-0644-8

**Published:** 2017-11-29

**Authors:** Qiaohua Peng, Jiale Qin, Yanan Zhang, Xiaodong Cheng, Xinyu Wang, Weiguo Lu, Xing Xie, Songfa Zhang

**Affiliations:** 1 Department of Gynecologic Oncology; Women’s Hospital, School of Medicine, Zhejiang University, Hangzhou, 310006 China; 2 Department of Ultrasound; Women’s Hospital; School of Medicine, Zhejiang University, Hangzhou, 310006 China; 3 Women’s Reproductive Health Laboratory of Zhejiang Province; Women’s Hospital; School of Medicine, Zhejiang University, Hangzhou, 310006 China

**Keywords:** Autophagy, Ovarian cancer, Cancer stem cell, FOXA2, Stemness

## Abstract

**Background:**

Cancer stem cells (CSCs) are regarded as the main cell type responsible for the initiation, metastasis, drug resistance, and recurrence of cancer. But the mechanism by which cancer stem cells maintain their stemness remains unclear.

**Methods and Results:**

In the present study, ovarian cancer stem cells (OCSCs) were revealed to have an enhanced autophagic flux. Furthermore, their chemoresistance and ability to self-renewal in vitro were decreased when autophagy was inhibited by Bafilomycin A1(BafA1), Chloroquine(CQ) or autophagy related 5(ATG5) knockdown. PCR array screening determined that Forkhead Box A2(FOXA2) was highly expressed in OCSCs, and correspondingly regulated by autophagy activity. In addition, the self-renewal ability was decreased in the case of FOXA2 knockdown by shRNA in OCSCs. Overexpression of FOXA2 from the pEGFP(+)-FOXA2 plasmid partially reversed the depressed self-renewal ability of OCSCs during autophagy inhibition.

**Conclusions:**

Our findings suggest that autophagy, through participation of FOXA2, maintains the characteristics of OCSCs. Autophagy and FOXA2 are therefore potential targets for ovarian cancer stem cell directed therapies.

**Electronic supplementary material:**

The online version of this article (10.1186/s13046-017-0644-8) contains supplementary material, which is available to authorized users.

## Background

Ovarian cancer is the most lethal malignancy in gynecological carcinomas, with an overall 5-year survival rate of only approximately 30–40%. The main factors driving this poor prognosis is the high rates of recurrence and drug resistance observed in this disease [1]. A growing number of recent studies have shown that CSCs are the source of relapse and chemoresistance of various cancers [2],and may serve as target cells for cancer therapeutics [3].

First identified in the hematopoietic system, CSCs have been successively identified in many different types of tumors (including ovarian cancer), by several putative markers, such as CD44, CD24, EpCAM, CD133, CD117, ALDH1 [4–6]. The CSCs theory hypothesizes that tumors may arise from a subpopulation of tumor cells endowed with self-renewal, tumorigenic, and multilineage differentiation capacities [7]. However, the mechanism by which CSCs maintain their stemness remains poorly understood.

Accumulating data show an intimate connection between autophagy and stemness [8].Autophagy involves the sequestration of cytoplasmic components within a double-membraned vesicle, and subsequent fusion with lysosomes to generate autolysosomes, in which the autophagic cargo is degraded by acidic hydrolases and recycled for cells to survive under various stress conditions [9]. Autophagy is essential to protect hematopoietic stem cells from metabolic stress and promotes survival in conditions of growth factor withdrawal and nutrient deprivation [10]. Loss of autophagy impairs hematopoietic stem-cell self-renewal activity and regenerative potential [11]. In addition, autophagy is also necessary for the maintenance of stemness of mesenchymal stem cells [12], muscle stem cells [13], and embryonic stem cells [14].More recently, it has been revealed that autophagy was upregulated in some CSCs, such as breast CSCs [15], pancreatic CSCs [16], and liver CSCs [17], and that compromising autophagy contributed to a decrease of self-renewal ability. However, both the mechanism by which autophagy maintains the stemness of CSCs and whether or not autophagy plays a critical role for ovarian CSCs (OCSCs) remain elusive.

In the present study, we enriched OCSCs from the ovarian cancer cell lines 3AO and SKOV3 using serum-free suspension culture system as we reported previously [18, 19], and observed greater autophagic flux in OCSCs compared to the bulk population of ovarian cancer cells. Furthermore, we demonstrate that the self-renewal ability and chemoresistance of OCSCs were decreased when autophagy was inhibited by BafA1, CQ or ATG5 knockdown. Using a stem cell signaling-related PCR array, we screened molecules that participated in maintaining the stemness of OCSCs, and verified that FOXA2 served as a stemness regulator in autophagy, controlling the self-renewal of OCSCs. The aim of our study was to investigate the role of autophagy and involved molecules in maintaining the stemness of OCSCs and possible targets for ovarian cancer therapeutics.

## Methods

### Cell culture and reagents

Ovarian adenocarcinoma cell line 3AO was obtained from the Women’s Hospital, School of Medicine, Zhejiang University. SKOV3 was obtained from the American Type Culture Collection (ATCC, HTB-77). 3AO and SKOV3 were maintained in RPMI 1640 (Corning, Steuben County, NY, USA) and McCoy’s 5A medium (Gibco, Carlsbad, CA, USA) respectively, supplemented with 10% fetal bovine serum. To form spheres, the 3AO and SKOV3 cells were cultured at a density of 50,000 cells/ml in serum-free DMEM/F12 medium (Cellgro, Virginia, USA) composed of 10 ng/ml basic fibroblast growth factor (bFGF, Peprotech, Rocky Hill, NJ, USA) and 20 ng/ml epidermal growth factor (EGF, Peprotech), 1 mg/ML insulin (Sigma–Aldrich, St Louis, MO, USA), and 1 × B27 supplement (Life Technologies,Carlsbad, CA, USA) on the ultralow attachplates (Corning, Steuben County, NY, USA). All cells were maintained in a humidified incubator with 5% CO_2_ at 37 °C. Nutrient deprivation (starvation) was induced through incubating cells in Earle’s Balanced Salt Solution (EBSS, Sigma–Aldrich); BafilomycinA1(BafA1, Sigma–Aldrich) and Chloroquine (CQ, Sigma–Aldrich) were dissolved in DMSO as stock solution. Paclitaxel was sourced from Bristol-Myers Squibb PharmaceuticalsLtd.

### RT^2^ profiler PCR arrays and qRT-PCR

We extracted total RNA from cells using TriZOL (Invitrogen, Carlsbad, CA, USA) according to themanufacturer’s instructions and further purified RNA using the RNeasy® MinElute™ Cleanup Kit (QIAGEN, Manchester, UK). RNA yield was then quantified via UV absorbance (NanoDrop® ND-1000) and quality was assessed by denaturing agarose gel electrophoresis to assess RNA, before generating cDNA by RT^2^ First Strand Kit (QIAGEN). Next, the cDNA is mixed with an appropriate RT^2^ SYBR Green Mastermix(QIAGEN, 330,601), aliquoted into the wells of the RT^2^ Profiler PCR Array (QIAGEN). Finally, relative expression was determined using the ∆∆CT method.

For qRT-PCR, RNA was extracted by TriZOL and reverse transcribed into cDNA using ReverTra Ace qPCR RT Kit (Toyobo, Japan). The subsequent quantitative RT-PCR was performed using Thunderbird SYBR qPCR Mix (Toyobo, Japan) and Applied Biosystems 7900HT fast real-time PCR System(Life Technologies, Carlsbad, CA, USA). GAPDH was used as an endogenous control for normalization. Primers sequences are listed in Additional file 1: Table S1.

### Western blotting analysis

Primary antibodies to FOXA2, NES(Nestin), NANOG(Nanog), POU5F1(Oct4), ALDH1A1(Aldehyde Dehydrogenase1 family member A1) and IL8(Interleukin 8) were purchased from Abcam. Antibodies to LC3B, GAPDH were obtained from Sigma–Aldrich, ATG5 and SQSTM1(sequestosome 1)were purchased from Cell Signaling Technology. Proteins were loaded and separated on SDS–PAGE (15% for LC3B; 10% for other proteins), then transferred to PVDF membranes(0.45-um, Corning). After blocking in 5% nonfat dry milk in TBST, membranes were incubated with primary antibodies overnight at 4°C and subsequent secondary antibodies. Membranes were then washed with TBST, and treated with EZ-ECL kit (BI biological industries, Cromwell, CT, USA) to detect bands in Imagequant LAS400mini (GE Healthcare). All experiments were performed at least three times.

### Flow Cytometry

To measure the expression of CD24 andCD44, cells were trypsinized into single cells, then washed twice with phosphate-buffered saline (PBS). Subsequently, 1 × 10^6^cells of 3AO were labeled with 1 unit of phycoerythrin mouse anti-humanCD24 (BD Bioscience Pharmingen Inc., San Diego, CA, USA) and 1 × 10^6^cells of SKOV3 were labeled with 1 unit of fluorescein isothiocyanate mouse anti-humanCD44 (BD Bioscience Pharmingen Inc.). Phycoerythrin Mouse IgG2a and Fluorescein isothiocyanate Mouse IgG2a were used as isotype controls (BD Bioscience Pharmingen Inc.). The surface expression of CD24 and CD44 were then assessed by flow cytometry (FCM, BD Bioscience Pharmingen Inc.) after incubation with antibody for 30 min at 4 °C in the dark.

### Plasmids and shRNA transfection

The pEGFP(+)-FOXA2 plasmidwas constructed as follows. The ORF encoding FOXA2 was synthesized by Sangon Biotech (Shanghai, China), and inserted into the EcoRI/BamHI sites of a pEGFP-N1 vector. We designated the empty pEGFP-N1 vector as the mock control. The GFP-LC3plasmid was a gift from Dr. Hong-He Zhang at Departmentof Pathology & Pathophysiology, School of Medicine, Zhejiang University (Hangzhou, China). SKOV3 and 3AO cells were grown to 80% confluency before plasmid transfection. The ratio of DNA:X-tremeGENE HP DNA Transfection Reagent (Roche, Basel, Switzerland) was 1:2, using 2 mg DNA for each well of a 6-well plate. The transfection protocol followed the instructions of the manufacturer. SKOV3 and 3AO cells were diluted to 10% to 15% confluencyafter a 24-h transfection of plasmids, then selected with 400 mg/mlG418 (Sigma–Aldrich) for 10 days.

Lentivirus vectors containing short hairpinRNAs against ATG5 and FOXA2, and scrambled control shRNA were obtained from Genechem(Shanghai, China). SKOV3 and 3AO cells were infected with the lentivirus vectors both at MOI 20 according to the manufacturer’s instructions. The expression of each protein was confirmed by Western blotting.

The sequences of shRNA are listed in Additional file 1: Table S1.

### Immunofluorescence analysis

3AO and SKOV3 cells were transfected with GFP-LC3 overnight and then transferred to coverslips. After adhering to coverslips, the cells were treated with 50 nM BafA1 or not for 4 h. The images were collected by using aspinning disk confocal fluorescence microscope (on a systemcomposed of a CSU-X1 spinning disk from Yokogama, a IX81microscope from Olympus(Tokyo, Japan) and a IXON3 CCD from Andor) at 600 × magnification. The Metamorph offline 7.7.8.0 software package was used to count the amounts of GFP-LC3-II-positivepuncta in 50 GFP-positive cells for each group.

### Transmission electron microscopy

TEM was conducted as previously described [20]. 3AO cells and 3AO CSCs were treated with 50 nM BafA1 or not for 4 h and fixed overnight with 2.5% glutaraldehyde solution (Sigma-Aldrich), postfixed with 1%OsO4 and then dehydrated standards were embedded in fractionated ethanol in812 resin (Ted Pella,Redding, CA, USA). The thin sections were sectioned and stained with 2% uranyl acetate and detected with a Tecnai 10 transmission electron microscope (Philips, Amsterdam, NED).

### Chemotherapy sensitivity assays

Sphere cells were seeded in ultra-low attachment 96-well plates at appropriate densities (9000 cells per well for SKOV3 and 8000 cells per well for 3AO), then exposed to paclitaxel at different final concentrations (0, 2, 5, 10, 20, 50 nM) for 48 h when autophagy was inhibited by ATG5 knockdown or treated with CQ (10 μM, 48 h).Each concentration was repeated in triplicate wells. Subsequently, Live cells was measured using CCK-8 kit (Dojindo laboratories, Kumamoto, Japan), and the absorbance was read by VarioskanFlash microplate reader (Thermo Scientific, Waltham, MA, USA). Cell viability was measured by ratio of absorbance value of paclitaxel treated/untreated wells in each group.

### Statistical analysis

Statistical analysis of differences between the groups was performed by using Student’s t-test. Results were presented as mean ± SD and a *P* value of less than 0.05 was considered to be significant.

## Results

### OCSCs were enriched from 3AO and SKOV3 ovarian cancer cells

**Fig. 1:**
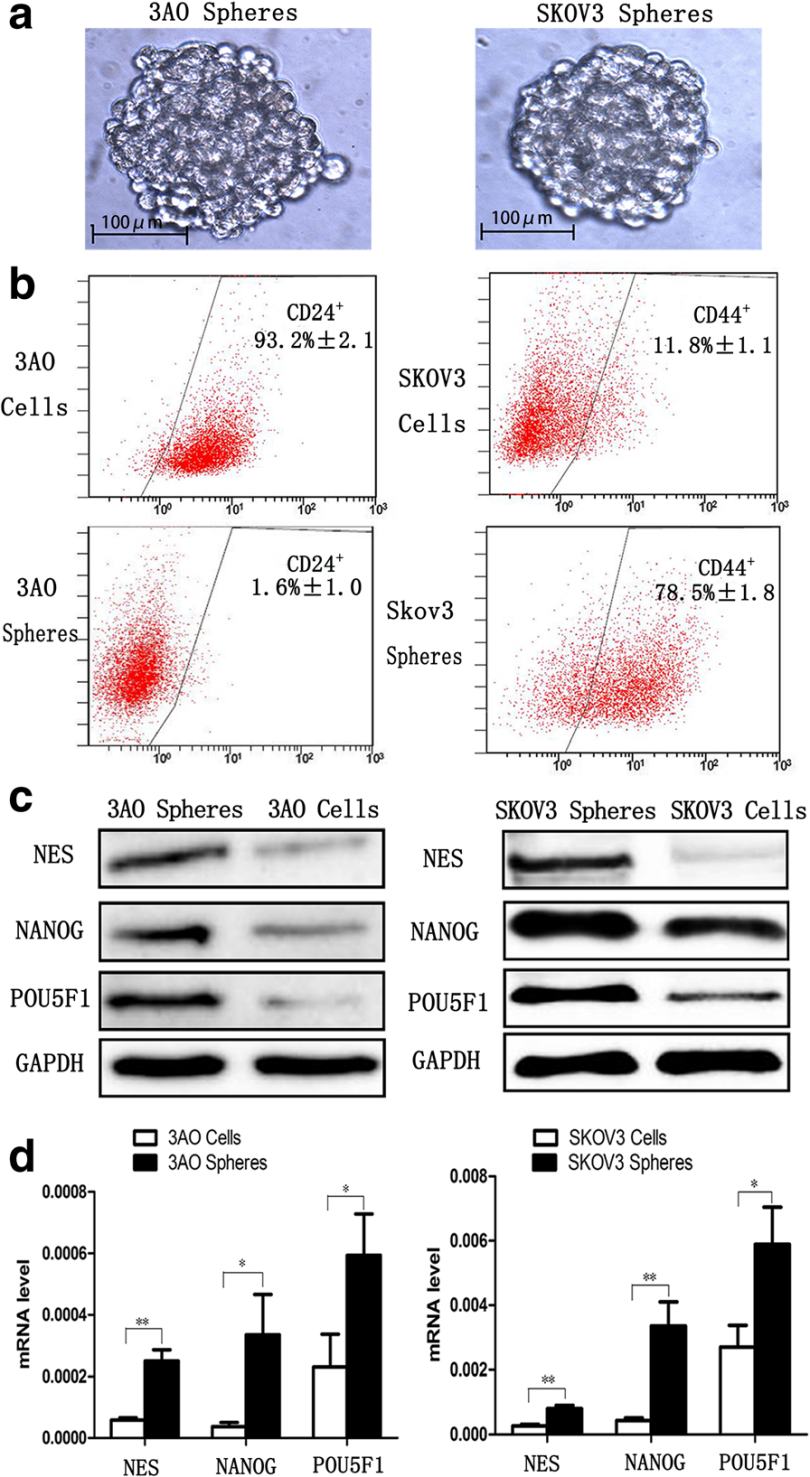
OCSCs were enriched from 3AO and SKOV3 ovarian cancer cells**. (a)**Spheres derived from 3AO and SKOV3 ovarian cancer cells maintained in serum-free medium culture system at 6 days (×200). **(b)** FCM (Flow Cytometry) analysis of CD24 and CD44 expression in 3AO and SKOV3 parental cells and sphere cells. **(c)** Protein expression of stemness regulators NES, NANOG, POU5F1 in 3AO and SKOV3 parental and sphere cells. **(d)** Messenger RNA expression of stemness regulators NES, NANOG, POU5F1 in 3AO, SKOV3 parental and sphere cells. Three independent experiments were performed and the results were expressed as the means ± SD, and analyzed using Student’s t-test (* *P* < 0.05, **P < 0.01)

Cancer cells grown in non-adherent cultures are able to form spherical clusters of cells (usually named spheres), which are rich of CSCs in vitro [18, 21].3AO and SKOV3 cells formed spheres after culture in a serum-free conditions for 6 days (Fig. 1a). FCM was used to identify the phenotype of sphere cells. The percentage of CD24^−^cells was 6.8% in the 3AO parental population cultured with 10%FBS, but this rose to a mean of 98.4% in cells from 3AO spheres averaging from three independent experiments (Fig.1b left). In SKOV3 spheres, the percentage of cells with a CD44^+^ phenotype – which is associated with stemness in ovarian cancer cells –increased to 78.5%, from 11.8% in the SKOV3 parental cells(Fig.1b right).To evaluate alterations of OCSCs percentage in spheres in further investigation, CD24 and CD44 were used to identify OCSCs in 3AO and SKOV3 spheres, respectively. Although we failed to found consistent surface markers to identify OCSCs in 3AO and SKOV3 spheres, mRNA and protein expressions of three stem cell regulators, NES (Nestin), NANOG (Nanog), and POU5F1 (Oct4), were all higher in 3AO and SKOV3 sphere cells than the parental cells(Fig.1c, Fig.1d), indicating enrichment of OCSCs in spheres.

### Autophagic flux was enhanced in OCSCs

**Fig. 2:**
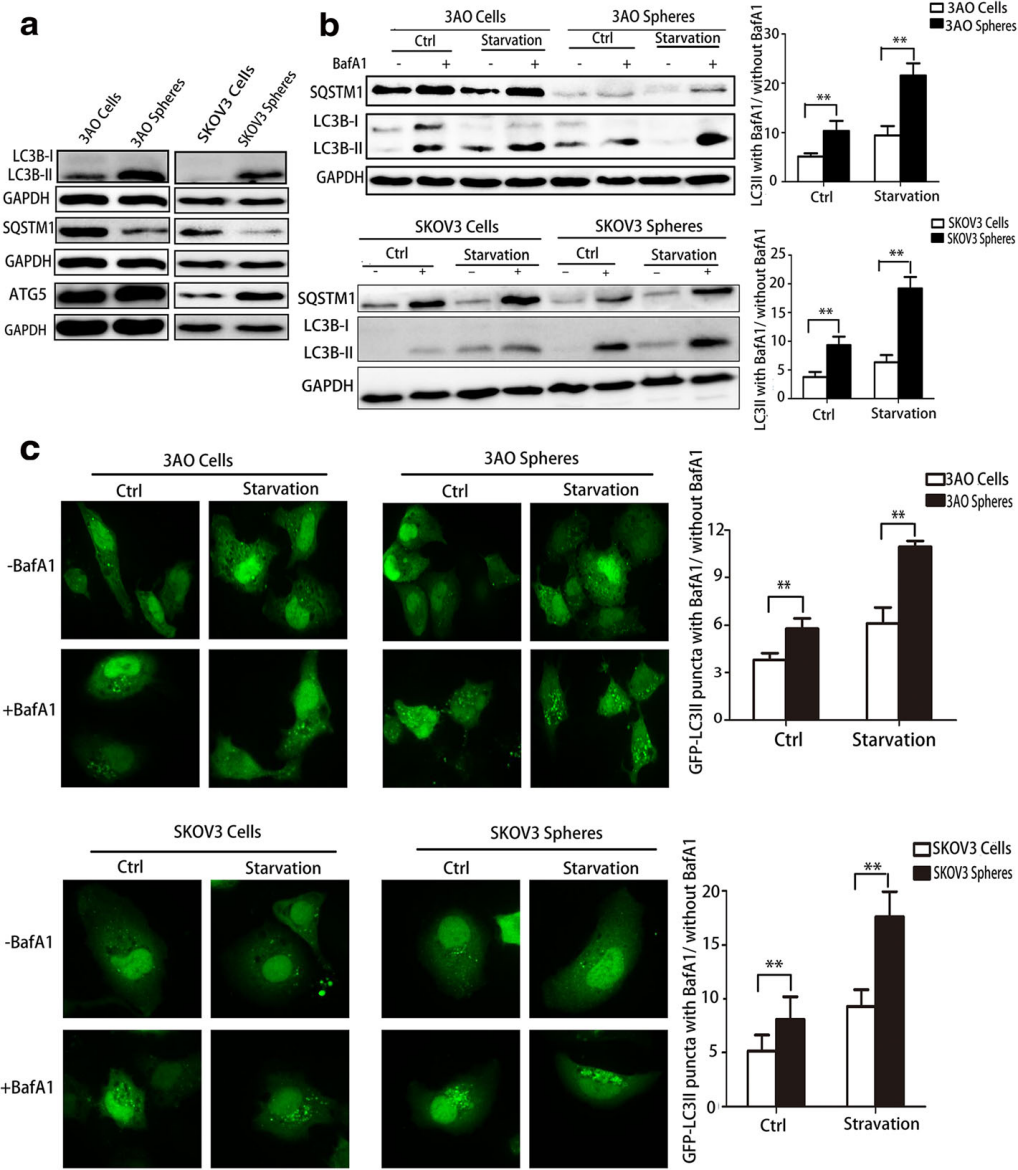
.The autophagic flux was enhanced in OCSCs. **(a)** Protein levels of LC3B, SQSTM1 and ATG5 were detected by Western Blotting in 3AO/SKOV3 parental and sphere cells. **(b)** Protein levels of SQSTM1 and LC3B were detected by Western Blotting in 3AO and SKOV3 parental and sphere cells. Cells were cultivated in complete medium (Ctrl) or starvation conditions (induced with EBSS for 4 h) in the presence or absence of BafA1 (final concentration 50 nM) for 4 h. GAPDH was analyzed as the loading control. The LC3B-II/GAPDH ratio was determined using the Quantity One software. The autophagic flux was determined as the ratio between the LC3B-II levels with and without BafA1 in histograms (right). **(c)**3AO-GFP-LC3B and SKOV3-GFP-LC3B parentaland sphere cells were cultivated as above, then visualized by confocal fluorescence microscopy. The number of GFP-LC3B-II positive puncta in 50 GFP-positive cells for each group was counted using the Metamorph offline 7.7.8.0 software package and the ratio between the GFP-LC3B-II-positive puncta per cell with BafA1 and without BafA1 was calculated (right). Three independent experiments were performed and the results were expressed as the means ± SD, and analyzed using Student’s t-test (* P < 0.05, **P < 0.01)

SQSTM1 (sequestosome 1) is an autophagy cargo protein that gets delivered to lysosomes for degradation, and ATG5 is essential for autophagosome formation. We determined the amount of SQSTM1 and ATG5, and found a higher rate of ATG5 production and SQSTM1degradation in 3AO and SKOV3 sphere cells than in parental cells (Fig.2a). The accumulation of LC3B-II (microtubule-associated protein 1 light chain 3B), the lipidated form of LC3B associated with the autophagosome membrane, was also increased in OCSCs (Fig.2a). Furthermore, BafA1, an inhibitor of vacular-type H^+^-ATPase that blocks lysosomal degradation, was used to demonstrate the autophagic flux. As indicated in Fig.2b, LC3B-II and SQSTM1 accumulated more in 3AO and SKOV3 sphere cells than those in parental cells in the presence of BafA1, regardless of the culture condition (i.e. under normal conditions with complete media or undergoing starvation in EBSS). Additionally, 3AO sphere cells were cultured in medium for spheres (serum-free) or for parental cells (with 10% fetal bovine serum) for 24 h, and there was no evident discrepancy of autophagic flux between these two groups (Additional file 2: Figure S1), indicating the increase of autophagic flux in sphere cells was not due to the nutrient conditions of spheres.

Confocal fluorescence microscopy was used to observe autophagosomes in 3AO and SKOV3 cells stably transfected with GFP-LC3. The number of GFP-LC3-II positive dots was increased in 3AO and SKOV3 sphere cells compared with parental cells, and further augmented in the presence of BafA1, especially in starvation conditions (Fig.2c). Furthermore, Transmission Election Microcopy(TEM) revealed that there was a greater number of larger autophagosomes and autolysosome vesicles in the 3AO sphere cells than in the bulk population, which became more pronounced in the presence of BafA1 (Additional file 3: FigureS2).

Taken together, these data suggest that OCSCs display robust autophagosome synthesis and an enhanced autophagic flux compared with their parental ovarian cancer cells, especially during the stress of nutrient deprivation.

### Autophagy is essential for maintaining of self-renewal and chemoresistance in OCSCs.

**Fig.3:**
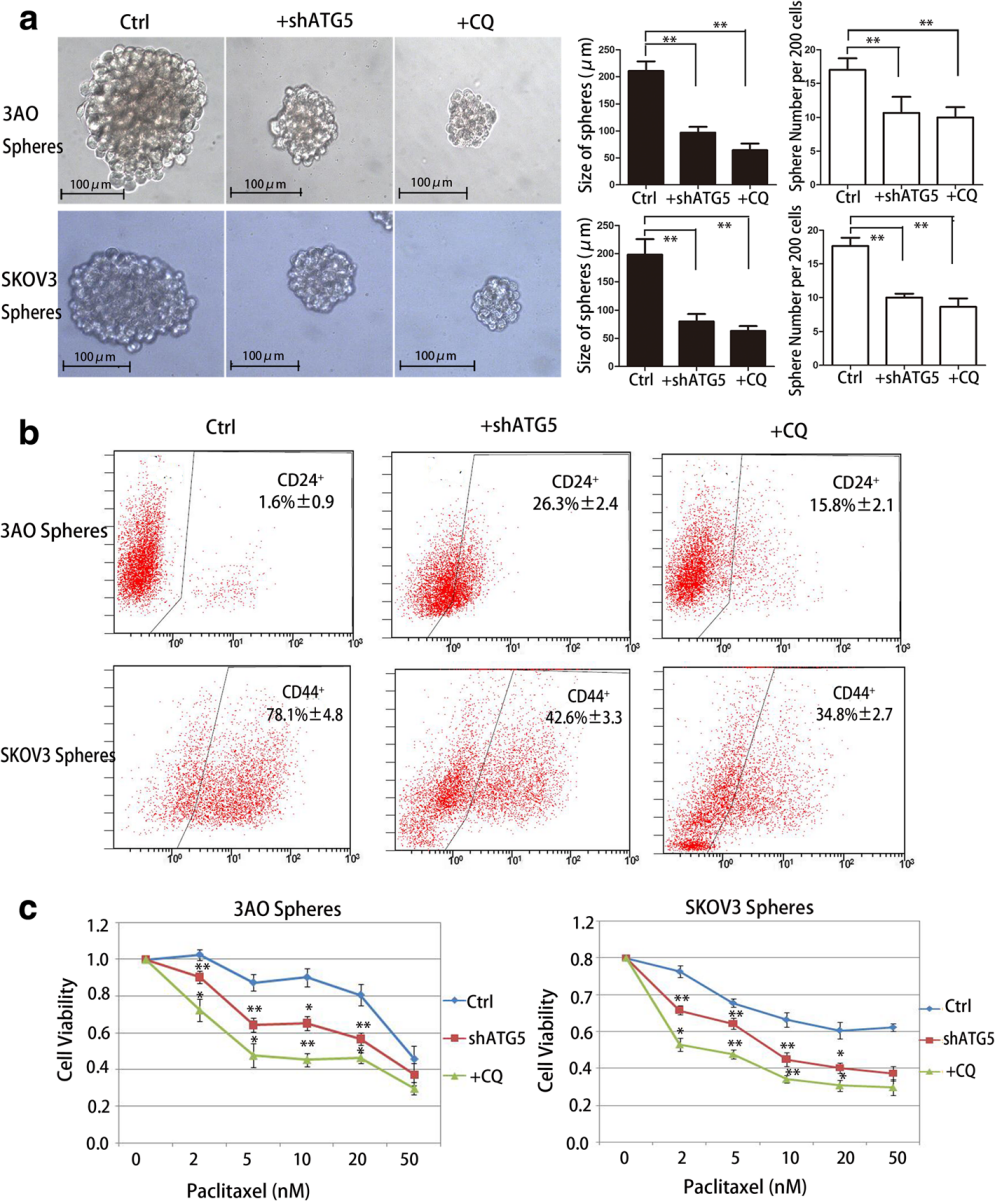
.Autophagy inhibition significantly reduces the characteristics of OCSCs. **(a)** 3AO and SKOV3 spheres were seeded at 200 cells per well in a 96-well ultralow attach plates and transfected with lentivirus expressing shRNA for ATG5 (shATG5) or scramble shRNA (Ctrl), or treated with 10 μM CQ (in the last 3 days). Spheres were observed through a microscope (left) and the size and number of the spheres were quantified after 6 days (right). **(b)** The CD24^−^ phenotype of 3AO spheres (upper) and the CD44^+^ phenotype of SKOV3 spheres (bottom) was determined by FCM after autophagy was inhibited by ATG5 knockdown or CQ(10 μM, 48 h). **(c)** 3AO and SKOV3 sphere cells were seeded in ultra-low attachment 96-well plates and exposed to paclitaxel at various concentrations for 48 h when autophagy was inhibited by ATG5 knockdown or treated with CQ (10 μM, 48 h). Live cells were measured using the CCK-8 kit. Cell viability was measured by ratio of absorbance value of paclitaxel treated/untreated wells in each group. Three independent experiments were performed and the results were expressed as the means ± SD, and analyzed using Student’s t-test (* *P* < 0.05, **P < 0.01)

The sphere formation assay was used to evaluate the self-renewal ability of OCSCs. The first generation of spheres were digested into single cells and reseeded. The size and number of the second generation of spheres were calculated after autophagy was inhibited with CQ or by ATG5 knockdown via shRNA lentivirus transfection. As shown in Additional file 4: Figure S3, autophagy suppression (*Fig.S3a*) and cytotoxic effect (*Fig.S3b*) were both increased following dose increase of CQ and BafA1. However, 10 μM CQ and 10 nM BafA1 could effectively suppress autophagy and cause no significant death in 3AO and SKOV3 cells. Additionally, 3AO and SKOV3 cells transfected with shATG5 lentivirus (MOI = 20) also did not increase cell death. We further measured cell viability in 3AO and SKOV3 sphere cells treated with 10 μM CQ and 10 nM BafA1 or by ATG5 knockdown, and no more cytotoxicity was found compared with control group (*Fig.S3c*).In spheres derived from the 3AO cell line(Fig.3a upper), the size (200 μm for control spheres versus 80 μm in the ATG5 knockdown, 50 μm when treated with CQ) and number (18 for the control versus 10 for ATG5 knockdown and 8 for CQ) of the second generation spheres formed from 200 single cells dramatically decreased upon inhibition of autophagy. Similar decreases were also found in spheres derived from SKOV3 cells (Fig.3a bottom).

An average of 98.4% of 3AO sphere cells were CD24^−^, indicating an OCSC phenotype, which was reduced to 73.7% and 84.2% when inhibiting autophagy by ATG5 knockdown or 10 μM CQ, respectively (Fig.3b upper). In SKOV3 sphere cells, the number of OCSCs with a CD44^+^ phenotype was reduced from 78.1% to 42.6% and 34.8% in the context of autophagy inhibition (Fig.3b bottom). In short, autophagy is essential not only for the formation of spheres, but also for enrichment of OCSCs in those spheres, reflecting the fact that autophagy can regulate the self-renewal ability of CSCs.

Chemoresistance is another feature of OCSCs, and autophagy is connected to paclitaxel resistance in ovarian cancer as we reported previously [20]. The influence of autophagy on paclitaxel sensitivity in OCSCs was further examined in the present study. 3AO and SKOV3 sphere cells were treated with different concentrations of paclitaxel (0, 2, 5, 10, 20 and 50 nM) for 48 h. After autophagy was attenuated, the number of surviving cells was significantly decreased compared with the control groups across different paclitaxel doses (Fig.3c). Our results demonstrate that inhibition of autophagy (by ATG5 knockdown or 48 h treatment with 10 μM CQ) significantly increased the sensitivity towards paclitaxel in3AO and SKOV3 derived OCSCs.

### FOXA2 is differentially expressed between OCSCs and parental ovarian cancer cells and is regulated by autophagy

**Fig. 4:**
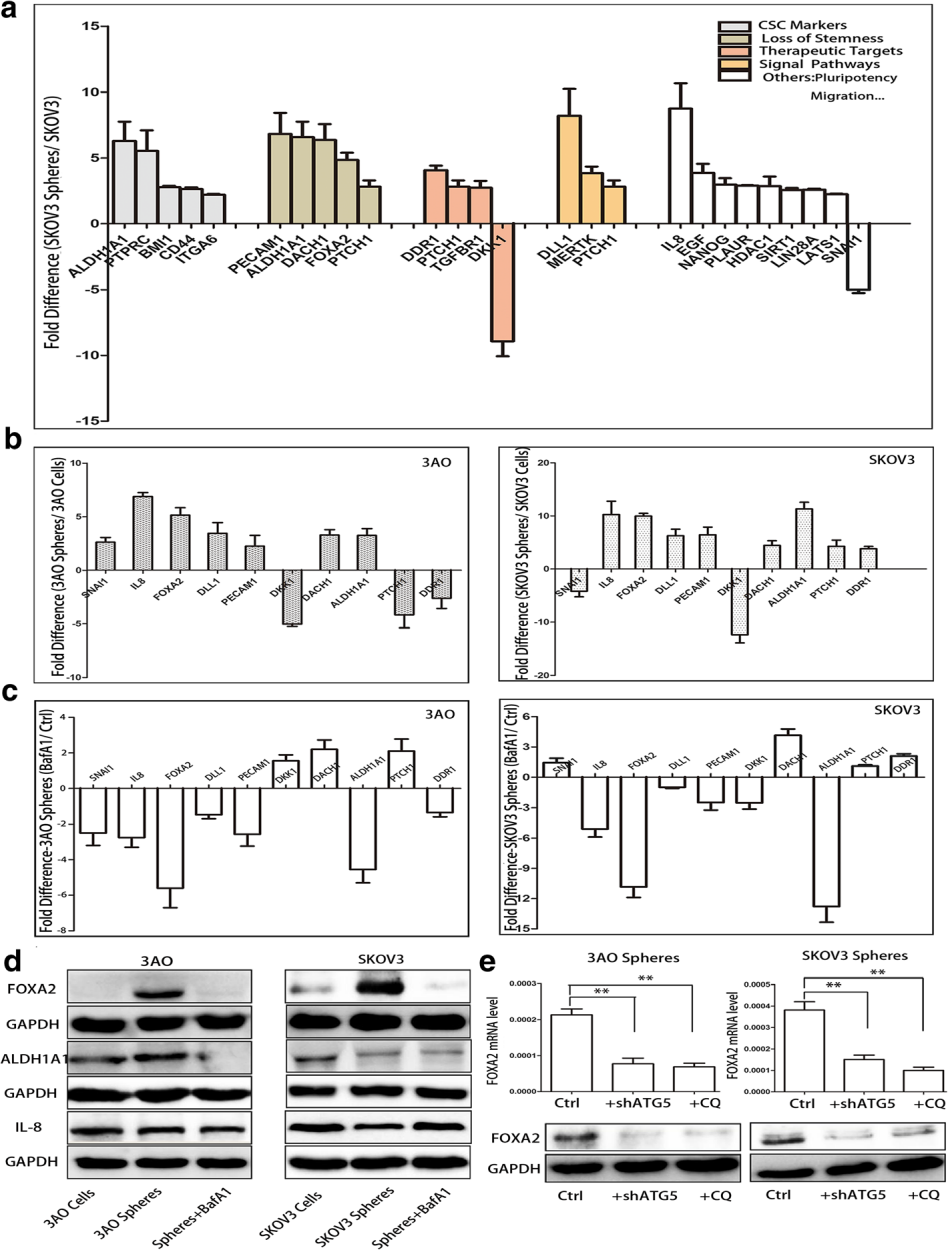
FOXA2 identified by PCR array was differentially expressed between spheres and parental cells and regulated by autophagy. **(a)** GO term enrichment analysis indicated that the 26 genes differentially expressed (more than 3-fold) could be categorized into cancer stem cell markers, loss of stemness, therapeutic targets, signal pathways, pluripotency, asymmetric division and migration. **(b)**Expression of 10 selected genes was validated between 3AO, SKOV3 parental and sphere cells by qRT-PCR. **(c)**Alteration of the 10 selected genes was determined using qRT-PCR after autophagy was inhibited using BafA1 in 3AO and SKOV3 sphere cells. **(d)** Protein levels of FOXA2, ALDH1A1, and IL8 were determined by Western Blotting in 3AO and SKOV3 parental and sphere cells treated with BafA1(10 nM, 48 h) or not. GAPDH was analyzed as the loading control. **(e)**The mRNA and protein levels of FOXA2 were measured in 3AO and SKOV3 spheres after autophagy was inhibited by ATG5 knockdown or CQ(10 μM, 48 h).Three independent experiments were performed and the results were expressed as the means ± SD, and analyzed using Student’s t-test (**P < 0.01)

The Human RT^2^ Profiler™ PCR Array was applied to profile the expression of 84 genes involved in stem cell signaling between SKOV3 parental cells and sphere cells. Of the 38 genes that displayed more than 2-fold differences between parental and sphere cells, 33 were upregulated and 5 were downregulated. Of these, 24 genes were upregulated more than 3-fold (maximum 8.8-fold) while 2 genes were downregulated more than 3-fold (maximum 9.4-fold) (Fig.4a). By cross-referencing these genes with the Gene Ontology (GO) term database, these 26 > 3-fold differentially expressed genes were categorized into cancer stem cell markers, loss of stemness, therapeutic targets, signal pathways, pluripotency, asymmetric division and migration.

To further confirm the alterations of gene expression revealed by PCR Array between 3AO and SKOV3 derived spheres and parental cells, 10 of the most differentially expressed genes (more than 5-fold) were selected for validation by qRT-PCR. All of the changes were consistent with the PCR Array results (Fig.4b). Furthermore, we used qRT-PCR to evaluate the expression of these 10 genes when autophagy was suppressed by BafA1 (10 nM, 48 h) in 3AO and SKOV3 sphere cells, and found that the high expression of FOXA2, IL8, and ALDH1A1 was significantly reduced after inhibiting autophagy (Fig.4c). Expression of the protein products of these genes was also assessed using Western Blotting. The level of FOXA2 protein was higher in spheres derived from 3AO and SKOV3 cells, and decreased after autophagy was inhibited by BafA1. However, comparable results were not observed for IL8 and ALDH1A1 under the same conditions (Fig.4d). Furthermore, we also found that levels of both mRNA and protein of FOXA2 were significantly decreased when autophagy was inhibited by CQ or ATG5 knockdown in 3AO and SKOV3 derived sphere cells (Fig.4e), suggesting that FOXA2 was regulated by autophagy.

Together, these findings imply that FOXA2 may be connected to autophagy and play a role in maintaining OCSCs stemness. Accordingly, FOXA2 was selected for further investigation.

### FOXA2 is required for the self-renewal ability of OCSCs

**Fig. 5:**
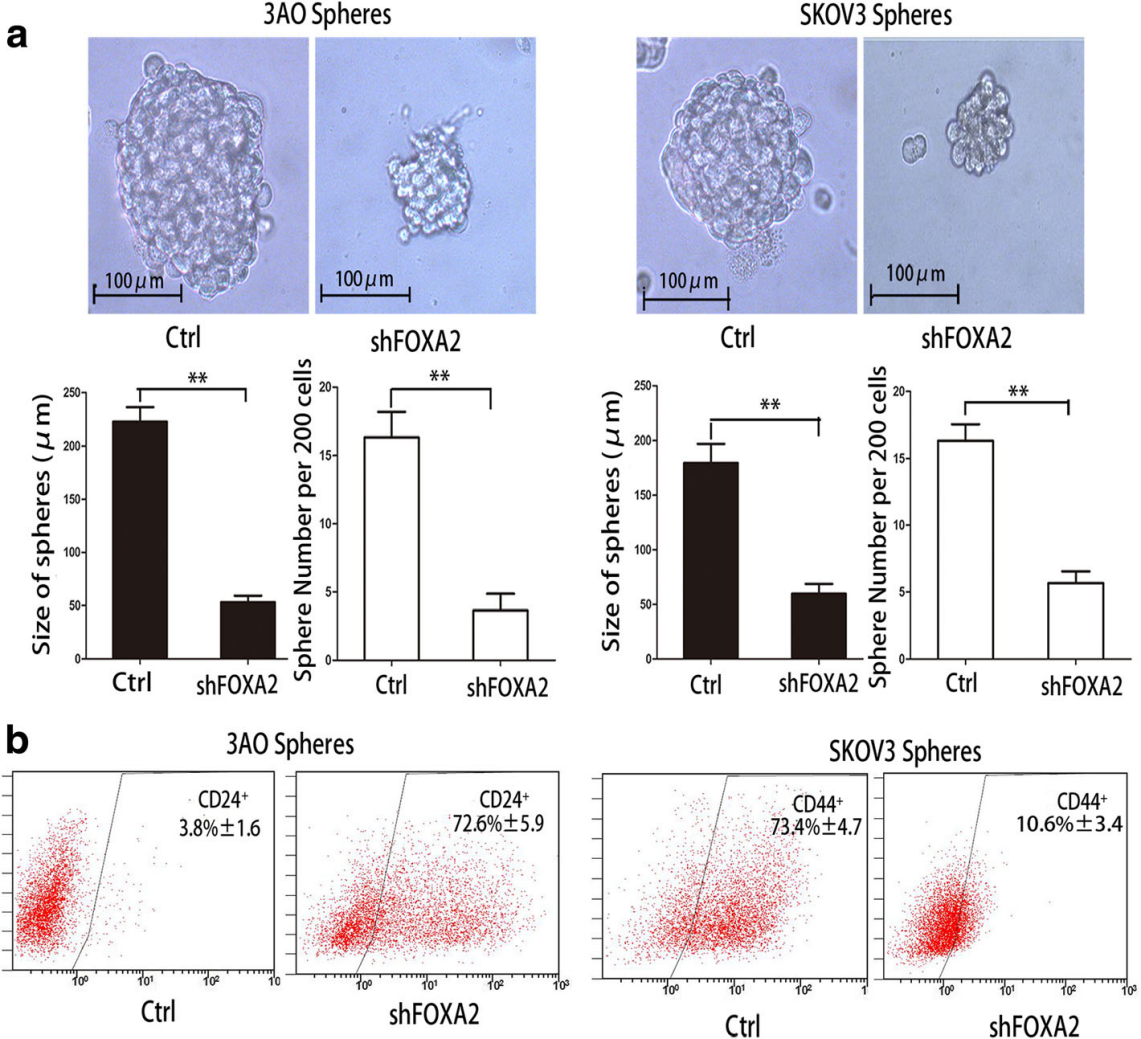
FOXA2 is required for self-renewal ability of OCSCs. 3AO and SKOV3 spheres were transfected with lentivirus expressing shRNA for FOXA2 (shFOXA2) or a scrambled shRNA (Ctrl). (**a**) Cells were seeded at 200 cells per well in a 96-well ultralow attach plates and grown for 6 days, and monitored under a microscope (upper); the size and number of the spheres were quantified (bottom). (**b**) The CD24^−^ phenotype of 3AO spheres (left) and the CD44^+^ phenotype of SKOV3 spheres (right) were determined by FCM. Three independent experiments were performed and data are shown asmeans ± SD(* *P* < 0.05, **P < 0.01)

We next further aimed to find out whether FOXA2 is critical for maintaining the self-renewal of OCSCs. As indicated in Fig.5a, the sphere formation assay revealed that when cloned from 200 single cells, the size of spheres (220 μm vs. 50 μm in 3AO; 185 μm vs. 55 μm in SKOV3) and their numbers (17 vs. 5 in 3AO; 17 vs. 6 in SKOV3) were significantly decreased when FOXA2 was knocked down using specific shRNA. Moreover, surface marker detection by FCM showed the percentage of CD24^−^OCSCs in 3AO spheres reduced from 96.2% to 27.4%, and the percentage of CD44^+^ OCSCs in SKOV3 spheres fell from 73.4% to 10.6% following FOXA2 knockdown (Fig.5b).

### Autophagy modulates self-renewal of OCSCs through FOXA2 participation

**Fig.6:**
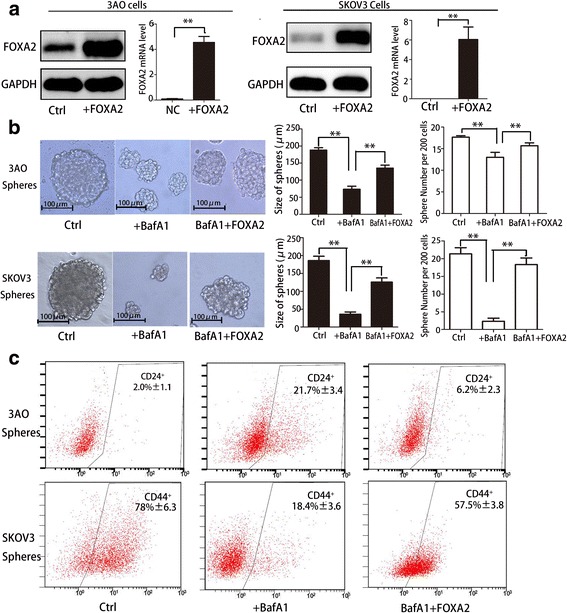
.Autophagy modulates the self-renewal of OCSCs through FOXA2 participation. **(a)** 3AO and SKOV3 cells were transfected with pEGFP(+)-FOXA2(+) or pEGFP(+)(Ctrl)plasmid and the FOXA2expression levels were assessed by Western Blotting and qRT-PCR. **(b)** The indicated cells were seeded at 200 cells per well in a 96-well ultralow attach plates and grown for 6 days, and were then treated with BafA1 (10 nM, 48 h) or not, and monitored under a microscope (left). The size and the number of the spheres were quantified (right). **(c)** Cells were treated and cultured as above; the CD24^−^ phenotype of 3AO spheres (upper) and the CD44^+^ phenotype of SKOV3 spheres (bottom) were determined by FCM. Three independent experiments were performed and the results were expressed as the means ± SD, and analyzed using Student’s t-test (* P < 0.05, **P < 0.01)

3AO and SKOV3 cells were transfected with pEGFP(+)-FOXA2(+) or pEGFP(+)plasmids. The results were verified through Western Blotting and qRT-PCR (Fig.6a). In 3AO derived spheres, the size of the spheres decreased from 180 μm to 55 μm after inhibiting autophagy with BafA1, which then reverted to 145 μm when FOXA2 was overexpressed. In line with this, the number of spheres cloned from 200 single cells reduced from 18 to 12 in conditions of autophagy suppression, and reversed to 16 after FOXA2 overexpression. Comparable results were also shown in SKOV3 derived spheres (Fig. 6b). In addition, sphere size and number were both partially reverted by FOXA2 overexpression when autophagy was suppressed by ATG5 knockdown in 3AO and SKOV3 sphere cells (Additional file 5: Figure S4). Furthermore, the cells possessing either a CD24^−^ (3AO spheres) or CD44^+^ (SKOV3 spheres) phenotype were both recovered by FOXA2 overexpression when autophagy was inhibited using BafA1 (Fig. 6c). Therefore, FOXA2 is involved in the pathway by which autophagy regulated self-renewal of OCSCs.

## Discussion

Cancer stem cells can be isolated from cancer cell lines and enriched in the form of spheres through serum-free suspension culture system. In the current study, we successfully isolated and enriched OCSCs from parental 3AO and SKOV3 cells utilizing the same method as we previously reported [18, 19]. There was however a difference in the surface markers expressed on 3AO and SKOV3 spheres. The former presented as CD24 negative while the latter were highly CD44 positive. Following our protocol, we were able to attain high percentages of OCSCs in spheres; 98.4% and 78.5% of CD24^−^ 3AO cells and CD44+ SKOV3 cells, respectively. Furthermore, some stemness regulators representing stem cell characteristics – including NES, NANOG, and POU5F1 – were all more highly expressed in CD24^−^ 3AO cells and CD44^+^ SKOV3 cells than in parental cells, suggesting that induction of sphere formation by serum-free suspension culture of 3AO and SKOV3 does indeed enrich cancer stem cells, which can be further studied.

A previous report showed that both autophagosome formation and autophagic flux were enhanced in mammospheres derived from breast cancer cell lines and primary tumors, while the size and number of mammospheres were decreased when autophagy was blocked by 3-MA, BafA1, CQ, or when essential autophagy genes, like ATG7 and BECN1, were knocked down [15]. Consistent results were also revealed in liver, pancreatic, and glioblastoma cancer stem cells [17, 22, 23]. Here, we demonstrate that the amount of both ATG5 and LC3B-II was markedly increased in 3AO and SKOV3 spheres, concurrent with increased degradation of the autophagy cargo protein SQSTM1, in the presence of BafA1 and in the context of starvation induced by EBSS treatment, compared with bulk ovarian cancer cells. These findings were confirmed by counting GFP-LC3-II positive puncta by confocal microscopy and visualization of autophagic vacuoles in TEM, suggesting that autophagic flux is increased in OCSCs. Further, we found remarkably decreased sphere formation capacity and a reduced percentage of cells bearing stemness markers in 3AO and SKOV3 sphere cells after autophagy was impeded by CQ and ATG5 knockdown, suggesting that an increase in autophagy is essential for maintaining the self-renewal of OCSCs. We also found paclitaxel sensitivity was dramatically increased in sphere cells when autophagy was suppressed by CQ and ATG5 knockdown. Because our previous findings suggested the self-renewal ability of sphere cells were suppressed after sphere cells were treated with CQ or by ATG5 knockdown for 6 days. To exclude the effect of self-renewal, we measured cell viability only after 2 days that sphere cells were exposed to paclitaxel with CQ and shATG5 lentivirus or not. Moreover, as shown in Additional file 4: Figure S3c, CQ or ATG5 knockdown did not cause extra cell death in sphere cells, suggesting the increase of paclitaxel sensitivity was due to inhibition of autophagy.

Some previous studies reported that several autophagy related genes – such as BECN1(Beclin 1, autophagy-related), ATG4A, ATG4C [15, 24, 25],ATM (Ataxia-Telangiectasia Mutated), and DDX53O (DEAD-box helicase 53) [25, 26]– were involved in its regulation and contributed to maintaining the stemness ofCSCs. However, those genes are more associated with the regulation of autophagy rather than with the link between autophagy and stemness. To uncover the molecules that are involved in maintaining stemness through modulation of autophagy in OCSCs, we applied a stem cell signaling related PCR array to screen for the expression of genes implicated in these processes. Of 38 differentially expressed genes, FOXA2 was highly expressed in 3AO and SKOV3 spheres and selected for further study.

FOXA2, a pioneer transcription factor and one of the members of Fox (Forkhead box) family, plays a key role in embryo development and is a key marker of endoderm cells [27]. Dysregulation of FOXA2 expression in fetal ventral mesencephalon-derived neural precursor cells appears to be associated with the loss of their potential to differentiate into dopaminergic neurons [28].FOXA2 is also used in the identification of a progenitor population that gives rise primarily to ventricular cardiovascular cells [29]. In addition, FOXA2 could maintain breast cancer stem cells, and downregulating it in these cells reduced the formation of spheres and the expression of CD44, ALDH1 [30]. Taken together, FOXA2 is a stemness regulator in normal stem cells and cancer stem cells. In the current study, we found that the size and number of spheres derived from 3AO and SKOV3 cells, and the percentage of cells bearing cancer stem cell phenotype markers in spheres, were all depressed after FOXA2 was knocked down, suggesting that FOXA2 modulates self-renewal of OCSCs. Furthermore, inhibition of autophagy suppressed FOXA2 expression, and more importantly, FOXA2 could revert the impairment of self-renewal capacity induced by compromising autophagy, suggesting that autophagy drives OCSCs maintenance through FOXA2.

It seems impossible that autophagy target FOXA2 through direct degradation, because our results showed inhibition of autophagy synchronously decrease mRNA and protein level of FOXA2. A few molecules or pathways may affect FOXA2, for instance, FOXA2 may be the target gene of transcription factors CREB1[31]and SOX17 [32], and can be epigenetically suppressed by hypoacetylation of histone H3 and H4 and trimethylation of H3K27 on the FOXA2 promoter [28]. Moreover, FOXA2 is a direct target gene of Hedgehog signaling pathway which involved in autophagy activation [33, 34]. But whether autophagy employs Hedgehog or other signaling pathways to regulate FOXA2 need to be carefully explored.

## Conclusions

In summary, autophagic flux is enhanced in OCSCs, and such enhanced autophagy facilitates the maintainence of stemness in OCSCs. Furthermore, the stemness-related transcription factor FOXA2 participates in this process. Our findings suggest that the modulation of autophagy may be a feasible approach to target cancer stem cells in potential ovarian cancer therapeutics.

## Additional files

Additional file 1: Table S1. Sequences of primer and shRNA. (DOC 37 kb)

Additional file 2: FigureS1. The serum-free condition did not affect basal autophagic flux of spheres. **(a)** Protein level of LC3B was detected by Western Blotting in 3AO sphere cells. Cells were cultivated in medium for spheres (serum-free) or medium for parental cells (with 10% fetal bovine serum) for 24 h in the presence or absence of BafA1. GAPDH was analyzed as the loading control. **(b)** The LC3B II/GAPDH ratio was determined by the Quentity One software. **(c)**The autophagic flux was determined as the ratio between the LC3B II levels with BafA1 and without BafA1 in histograms. Three independent experiments were performed and the results were expressed as the means ± SD, and analyzed using Student’s t-test. (TIFF 402 kb)

Additional file 3: Figure S2 . Autophagic vesicles were visualized by transmission electron microscopy. Autophagosome and autolysosome vesicles were visualized by transmission electron microcopy in 3AO and sphere cells treated with BafA1 (50 nM, 4 h) or not. The typical images of autophagic vesicles (red arrows) were shown at high magnification. (TIFF 6804 kb)

Additional file 4: Figure S3. Blockage of autophagy by shATG5, CQ or BafA1 in our working conditions did not increase cell death. **(a)** 3AO and SKOV3 cells were treated with different concentrations of CQ (0, 2, 5, 10, 20 μM) or BafA1 (0, 2, 5, 10, 20 nM) for 48 h. Protein level of LC3B was detected by Western Blotting. GAPDH was analyzed as the loading control. **(b)** 3AO and SKOV3 cells were transfected with shATG5 lentivirus (MOI = 20), or treated with different concentrations of CQ (10 and 20 μM) or BafA1 (10 and 20 nM) for 48 h. Live cells were measured by Trypan Blue staining. **(c)** 3AO and SKOV3 spheres were treated with shATG5 lentivirus (MOI = 20), CQ (10 μM), or BafA1 (10 nM) for 48 h, Live cells was measured by Trypan Blue staining. Three independent experiments were performed and the results were expressed as the means ± SD, and analyzed using Student’s t-test (* *P* < 0.05, **P < 0.01). (TIFF 4132 kb)

Additional file 5: Figure S4. Self-renewal ability of OCSCs decreased by ATG5 silencing can be partly reverted by FOXA2 overexpression. 3AO and SKOV3 spheres transfected with pEGFP(+)-FOXA2(+) or pEGFP(+) (Ctrl) plasmid after transfection with shATG5 lentivirus. Cells were seeded at 200 cells per well in a 96-well ultralow attach plate and grew for 6 days, then observed the formation of spheres under a microscope (left). The size and the number of the spheres were also quantified (right). Three independent experiments were performed and the results were expressed as the means ± SD, and analyzed using Student’s t-test (* P < 0.05, **P < 0.01). (TIFF 4277 kb)

## Abbreviations

ALDH1A1: Aldehyde Dehydrogenase1 family, member A1
ATG5: autophagy related 5
BafA1: BafilomycinA1
BECN1: Beclin1, autophagy-related
bFGF: basic fibroblast growth factor
CQ: Chloroquine
CSCs: Cancer stem cells
EBSS: Earle’s Balanced Salt Solution
EGF: epidermal growth factor
FOXA2: Forkhead Box A2
GO: gene ontology
IL8: Interleukin 8
MAP1LC3B (LC3B): microtubule-associated protein 1 light chain 3B
OCSCs: Ovarian CSCs
SQSTM1: sequestosome 1

## References

[1] Gangemi R, Paleari L, Orengo AM, Cesario A, Chessa L, Ferrini S, Russo P (2009). Cancer stem cells: a new paradigm for understanding tumor growth and progression and drug resistance. Curr Med Chem.

[2] Clarke MF, Dick JE, Dirks PB, Eaves CJ, Jamieson CH, Jones DL, Visvader J, Weissman IL, Wahl GM (2006). Cancer stem cells--perspectives on current status and future directions: AACR workshop on cancer stem cells. Cancer Res.

[3] Zhou BB, Zhang H, Damelin M, Geles KG, Grindley JC, Dirks PB (2009). Tumour-initiating cells: challenges and opportunities for anticancer drug discovery. Nat Rev Drug Discov.

[4] Clevers H (2011). The cancer stem cell: premises, promises and challenges. Nat Med.

[5] Meng E, Long B, Sullivan P, McClellan S, Finan MA, Reed E, Shevde L, Rocconi RP (2012). CD44+/CD24- ovarian cancer cells demonstrate cancer stem cell properties and correlate to survival. Clinical & experimental metastasis.

[6] Wang J, Chen D, He X, Zhang Y, Shi F, Wu D, Chen J, Zhang Y, Zhao F, Dou J (2015). Downregulated lincRNA HOTAIR expression in ovarian cancer stem cells decreases its tumorgeniesis and metastasis by inhibiting epithelial-mesenchymal transition. Cancer Cell Int.

[7] Lobo NA, Shimono Y, Qian D, Clarke MF (2007). The biology of cancer stem cells. Annu Rev Cell Dev Biol.

[8] Pan H, Cai N, Li M, Liu GH, Izpisua Belmonte JC (2013). Autophagic control of cell 'stemness. EMBO molecular medicine.

[9] Galluzzi L, Pietrocola F, Levine B, Kroemer G (2014). Metabolic control of autophagy. Cell.

[10] Warr MR, Binnewies M, Flach J, Reynaud D, Garg T, Malhotra R, Debnath J, Passegue E (2013). FOXO3A directs a protective autophagy program in haematopoietic stem cells. Nature.

[11] Ho TT, Warr MR, Adelman ER, Lansinger OM, Flach J, Verovskaya EV, Figueroa ME, Passegue E (2017). Autophagy maintains the metabolism and function of young and old stem cells. Nature.

[12] Sbrana FV, Cortini M, Avnet S, Perut F, Columbaro M, De Milito A, Baldini N (2016). The role of autophagy in the maintenance of Stemness and differentiation of Mesenchymal stem cells. Stem Cell Rev.

[13] Garcia-Prat L, Martinez-Vicente M, Perdiguero E, Ortet L, Rodriguez-Ubreva J, Rebollo E, Ruiz-Bonilla V, Gutarra S, Ballestar E, Serrano AL (2016). Autophagy maintains stemness by preventing senescence. Nature.

[14] Liu P, Liu K, Gu H, Wang W, Gong J, Zhu Y, Zhao Q, Cao J, Han C, Gao F, et al. High autophagic flux guards ESC identity through coordinating autophagy machinery gene program by FOXO1. Cell Death Differ. 2017; 10.1038/cdd.2017.90 PMC5596427 28622295

[15] Gong C, Bauvy C, Tonelli G, Yue W, Delomenie C, Nicolas V, Zhu Y, Domergue V, Marin-Esteban V, Tharinger H *et al*: Beclin 1 and autophagy are required for the tumorigenicity of breast cancer stem-like/progenitor cells. *Oncogene* 2013, 32(18):2261–2272, 2272e 2261–2211. 10.1038/onc.2012.252 PMC3679409 22733132

[16] Rausch V, Liu L, Apel A, Rettig T, Gladkich J, Labsch S, Kallifatidis G, Kaczorowski A, Groth A, Gross W (2012). Autophagy mediates survival of pancreatic tumour-initiating cells in a hypoxic microenvironment. J Pathol.

[17] Song YJ, Zhang SS, Guo XL, Sun K, Han ZP, Li R, Zhao QD, Deng WJ, Xie XQ, Zhang JW (2013). Autophagy contributes to the survival of CD133+ liver cancer stem cells in the hypoxic and nutrient-deprived tumor microenvironment. Cancer Lett.

[18] Shi MF, Jiao J, WG L, Ye F, Ma D, Dong QG, Xie X (2010). Identification of cancer stem cell-like cells from human epithelial ovarian carcinoma cell line. Cellular and molecular life sciences : CMLS.

[19] Huang L, Xu S, Hu D, Lu W, Xie X, Cheng X (2015). IQGAP1 is involved in enhanced aggressive behavior of epithelial ovarian cancer stem cell-like cells during differentiation. International journal of gynecological cancer : official journal of the International Gynecological Cancer Society.

[20] Zhang SF, Wang XY, ZQ F, Peng QH, Zhang JY, Ye F, YF F, Zhou CY, WG L, Cheng XD (2015). TXNDC17 promotes paclitaxel resistance via inducing autophagy in ovarian cancer. Autophagy.

[21] Ponti D, Costa A, Zaffaroni N, Pratesi G, Petrangolini G, Coradini D, Pilotti S, Pierotti MA, Daidone MG (2005). Isolation and in vitro propagation of tumorigenic breast cancer cells with stem/progenitor cell properties. Cancer Res.

[22] Zhu H, Wang D, Zhang L, Xie X, Wu Y, Liu Y, Shao G, Su Z (2014). Upregulation of autophagy by hypoxia-inducible factor-1alpha promotes EMT and metastatic ability of CD133+ pancreatic cancer stem-like cells during intermittent hypoxia. Oncol Rep.

[23] Galavotti S, Bartesaghi S, Faccenda D, Shaked-Rabi M, Sanzone S, McEvoy A, Dinsdale D, Condorelli F, Brandner S, Campanella M (2013). The autophagy-associated factors DRAM1 and p62 regulate cell migration and invasion in glioblastoma stem cells. Oncogene.

[24] Wolf J, Dewi DL, Fredebohm J, Muller-Decker K, Flechtenmacher C, Hoheisel JD, Boettcher M (2013). A mammosphere formation RNAi screen reveals that ATG4A promotes a breast cancer stem-like phenotype. Breast cancer research : BCR.

[25] Antonelli M, Strappazzon F, Arisi I, Brandi R, D'Onofrio M, Sambucci M, Manic G, Vitale I, Barila D, Stagni V (2017). ATM kinase sustains breast cancer stem-like cells by promoting ATG4C expression and autophagy. Oncotarget.

[26] Kim H, Kim Y, Jeoung D (2017). DDX53 promotes cancer stem cell-like properties and autophagy. Molecules and cells.

[27] Mfopou JK, Geeraerts M, Dejene R, Van Langenhoven S, Aberkane A, Van Grunsven LA, Bouwens L (2014). Efficient definitive endoderm induction from mouse embryonic stem cell adherent cultures: a rapid screening model for differentiation studies. Stem Cell Res.

[28] Bang SY, Kwon SH, Yi SH, Yi SA, Park EK, Lee JC, Jang CG, You JS, Lee SH, Han JW (2015). Epigenetic activation of the Foxa2 gene is required for maintaining the potential of neural precursor cells to differentiate into dopaminergic neurons after expansion. Stem Cells Dev.

[29] Bardot E, Calderon D, Santoriello F, Han S, Cheung K, Jadhav B, Burtscher I, Artap S, Jain R, Epstein J (2017). Foxa2 identifies a cardiac progenitor population with ventricular differentiation potential. Nat Commun.

[30] Perez-Balaguer A, Ortiz-Martinez F, Garcia-Martinez A, Pomares-Navarro C, Lerma E, Peiro G (2015). FOXA2 mRNA expression is associated with relapse in patients with triple-negative/basal-like breast carcinoma. Breast Cancer Res Treat.

[31] Baroukh N, Ravier MA, Loder MK, Hill EV, Bounacer A, Scharfmann R, Rutter GA, Van Obberghen E (2007). MicroRNA-124a regulates Foxa2 expression and intracellular signaling in pancreatic beta-cell lines. J Biol Chem.

[32] Sinner D, Rankin S, Lee M, Zorn AM (2004). Sox17 and beta-catenin cooperate to regulate the transcription of endodermal genes. Development.

[33] Wang DH, Tiwari A, Kim ME, Clemons NJ, Regmi NL, Hodges WA, Berman DM, Montgomery EA, Watkins DN, Zhang X (2014). Hedgehog signaling regulates FOXA2 in esophageal embryogenesis and Barrett's metaplasia. J Clin Invest.

[34] Jimenez-Sanchez M, Menzies FM, Chang YY, Simecek N, Neufeld TP, Rubinsztein DC (2012). The hedgehog signalling pathway regulates autophagy. Nat Commun.

