# Supplementary material for: Autophagy maintains the stemness of ovarian cancer stem cells by FOXA2
Source: J Exp Clin Cancer Res. 2017 Nov 29;36:171. doi: 10.1186/s13046-017-0644-8 (PMC5707869; doi:10.1186/s13046-017-0644-8)
Supplement: Supplementary file 1 — Sequences of primer and shRNA. (DOC 37 kb) [file 13046_2017_644_MOESM1_ESM.doc]

**Supplemental Table S1: Sequences of primer and shRNA.**

| Gene Accession NO. | Sequence(5’to 3’) |
| --- | --- |
| SNAI1 | ACTGCAACAAGGAATACCTCAG |
|  | GCACTGGTACTTCTTGACATCTG |
| IL8 | GTGAGTTTGTTGTACTCATGACCAG |
|  | GGCACAGTGGAACAAGGACT |
| FOXA2 | GGAGCAGCTACTATGCAGAGC |
|  | CGTGTTCATGCCGTTCATCC |
| DLL1 | GACGAACACTACTACGGAGAGG |
|  | AGCCAGGGTTGCACACTTT |
| PECAM1 | AACAGTGTTGACATGAAGAGCC |
|  | TGTAAAACAGCACGTCATCCTT |
| DKK1 | CCTTGAACTCGGTTCTCAATTCC |
|  | CAATGGTCTGGTACTTATTCCCG |
| DACH1 | ATGTGGAACAAGTTCGCATCC |
|  | TGCAGTCATTGTAGAGGGTCT |
| ALDH1A1 | CTGCTGGCGACAATGGAGT |
|  | CGCAATGTTTTGATGCAGCCT |
| PTCH1 | GCCATGAATGTTGAGAAAGCCT |
|  | GTCCTGGACACTATCTTCAGAGT |
| DDR1 | CCGACTGGTTCGCTTCTACC |
|  | CGGTGTAAGACAGGAGTCCATC |
| FOXA2 shRNA | CTCCTCTTAAGAAGACGAC |
| ATG5 shRNA | TTTCATTCAGAAGCTGTTT |
