# Supplementary figures and images for: Autophagy maintains the stemness of ovarian cancer stem cells by FOXA2
Source: J Exp Clin Cancer Res. 2017 Nov 29;36:171. doi: 10.1186/s13046-017-0644-8 (PMC5707869; doi:10.1186/s13046-017-0644-8)

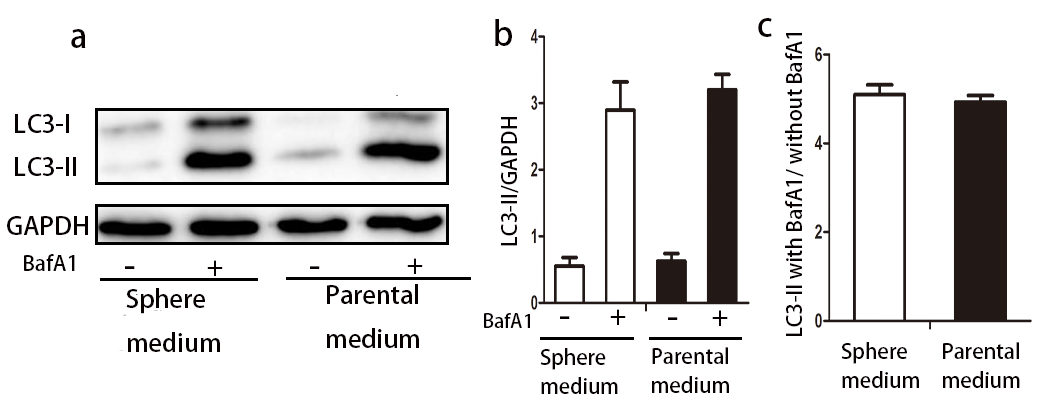

Supplement: Supplementary file 2 — The serum-free condition did not affect basal autophagic flux of spheres. (a) Protein level of LC3B was detected by Western Blotting in 3AO sphere cells. Cells were cultivated in medium for spheres (serum-free) or medium for parental cells (with 10% fetal bovine serum) for 24 h in the presence or absence of BafA1. GAPDH was analyzed as the loading control. (b) The LC3B II/GAPDH ratio was determined by the Quentity One software. (c)The autophagic flux was determined as the ratio between the LC3B II levels with BafA1 and without BafA1 in histograms. Three independent experiments were performed and the results were expressed as the means ± SD, and analyzed using Student’s t-test. (TIFF 402 kb) [file 13046_2017_644_MOESM2_ESM.tif]

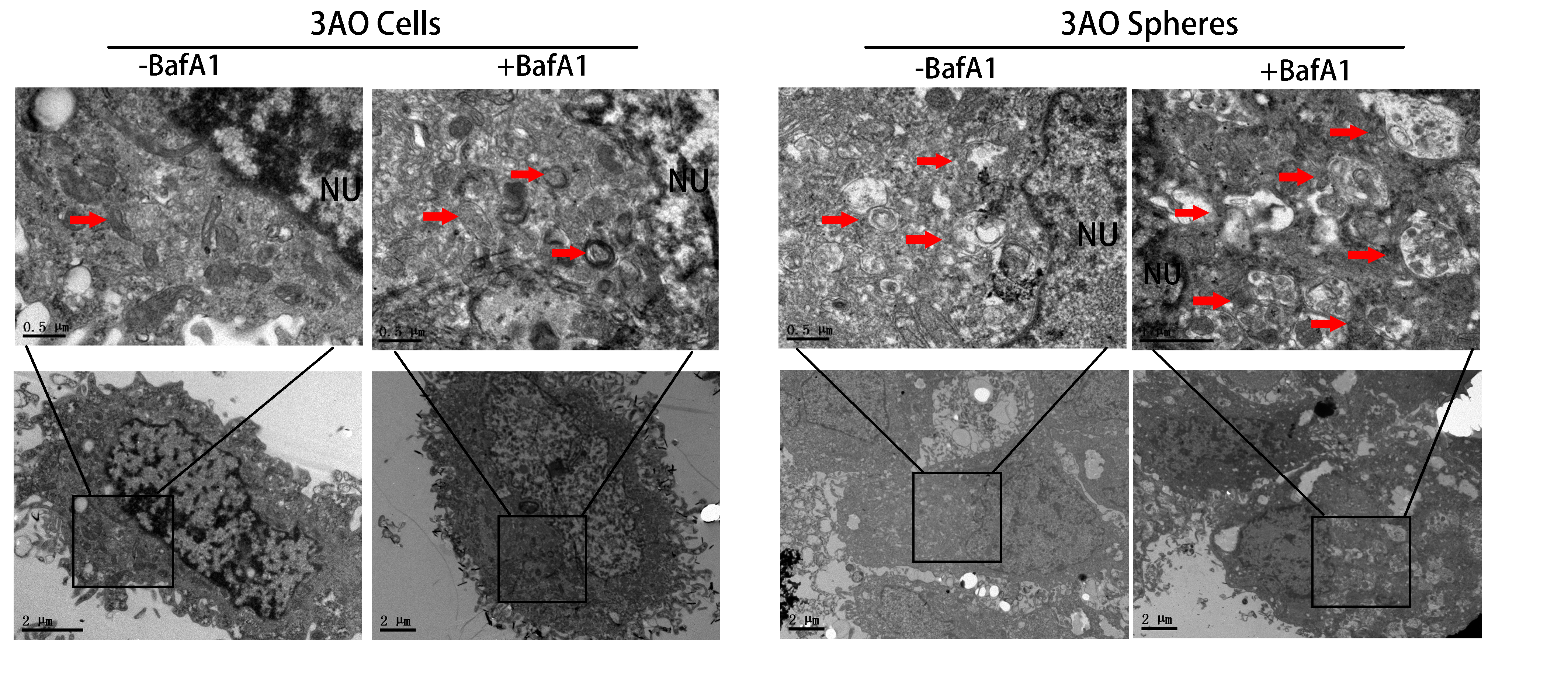

Supplement: Supplementary file 3 — Autophagic vesicles were visualized by transmission electron microscopy. Autophagosome and autolysosome vesicles were visualized by transmission electron microcopy in 3AO and sphere cells treated with BafA1 (50 nM, 4 h) or not. The typical images of autophagic vesicles (red arrows) were shown at high magnification. (TIFF 6804 kb) [file 13046_2017_644_MOESM3_ESM.tif]

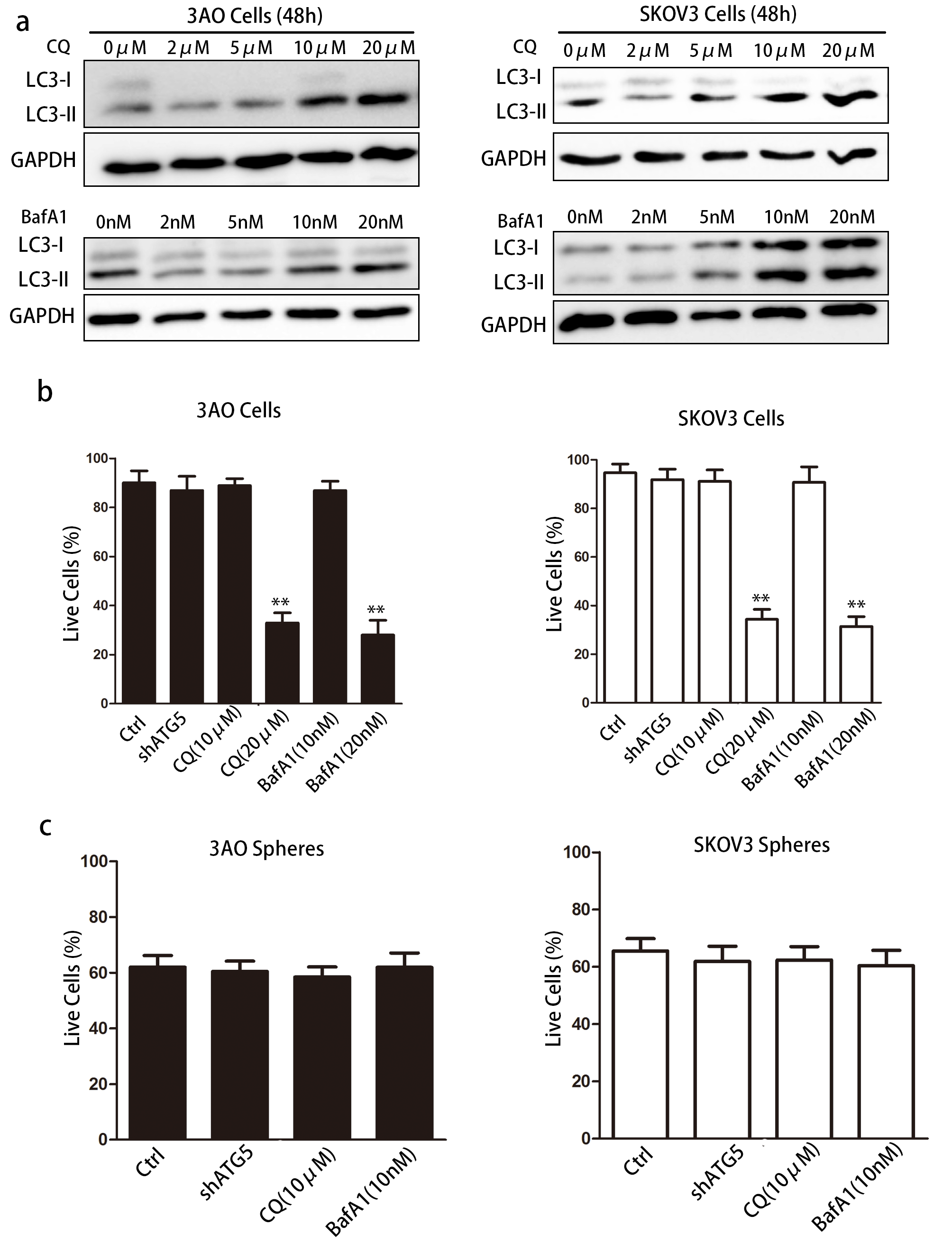

Supplement: Supplementary file 4 — Blockage of autophagy by shATG5, CQ or BafA1 in our working conditions did not increase cell death. (a) 3AO and SKOV3 cells were treated with different concentrations of CQ (0, 2, 5, 10, 20 μM) or BafA1 (0, 2, 5, 10, 20 nM) for 48 h. Protein level of LC3B was detected by Western Blotting. GAPDH was analyzed as the loading control. (b) 3AO and SKOV3 cells were transfected with shATG5 lentivirus (MOI = 20), or treated with different concentrations of CQ (10 and 20 μM) or BafA1 (10 and 20 nM) for 48 h. Live cells were measured by Trypan Blue staining. (c) 3AO and SKOV3 spheres were treated with shATG5 lentivirus (MOI = 20), CQ (10 μM), or BafA1 (10 nM) for 48 h, Live cells was measured by Trypan Blue staining. Three independent experiments were performed and the results were expressed as the means ± SD, and analyzed using Student’s t-test (* P < 0.05, **P < 0.01). (TIFF 4132 kb) [file 13046_2017_644_MOESM4_ESM.tif]

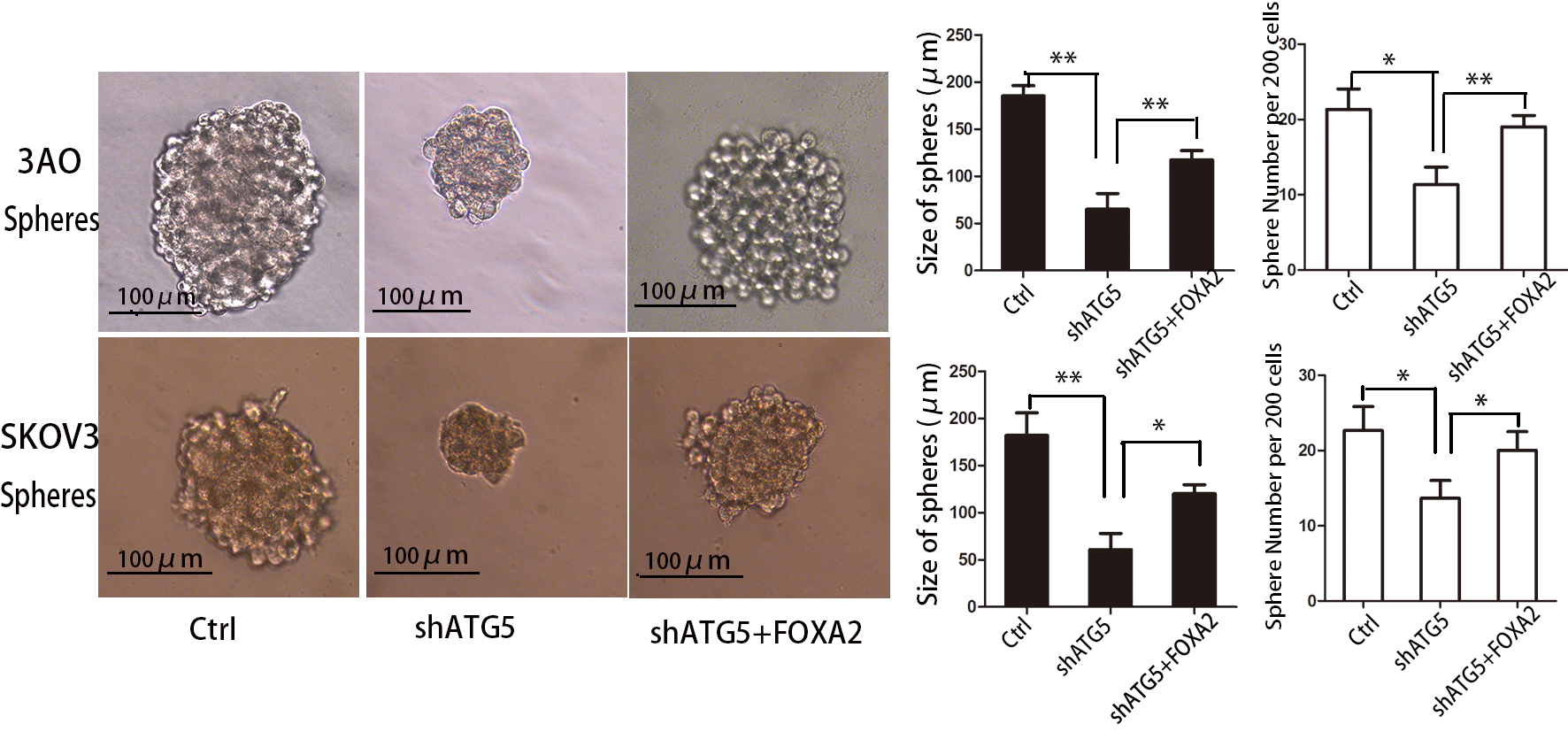

Supplement: Supplementary file 5 — Self-renewal ability of OCSCs decreased by ATG5 silencing can be partly reverted by FOXA2 overexpression. 3AO and SKOV3 spheres transfected with pEGFP(+)-FOXA2(+) or pEGFP(+) (Ctrl) plasmid after transfection with shATG5 lentivirus. Cells were seeded at 200 cells per well in a 96-well ultralow attach plate and grew for 6 days, then observed the formation of spheres under a microscope (left). The size and the number of the spheres were also quantified (right). Three independent experiments were performed and the results were expressed as the means ± SD, and analyzed using Student’s t-test (* P < 0.05, **P < 0.01). (TIFF 4277 kb) [file 13046_2017_644_MOESM5_ESM.tif]
